# Fluorescence-aided stereotactic needle biopsies for brain tumors: experience on 516 cases

**DOI:** 10.1007/s10072-026-09032-1

**Published:** 2026-04-15

**Authors:** Morgan Broggi, Isabella Minotti, Jacopo Falco, Marco Schiariti, Francesco Restelli, Massimiliano Del Bene, Edoardo Porto, Federico Legnani, Francesco Acerbi, Francesco DiMeco, Paolo Ferroli

**Affiliations:** 1 Department of Neurosurgery, Fondazione IRCCS Istituto Neurologico Carlo Besta, Via G. Celoria 11, 20133 Milan, Italy; 2 Neurosurgery Unit, Pisa University Hospital, Pisa, Italy; 3 Department of Translational Research and New Technology in Medine and Surgery, University of Pisa, Pisa, Italy

**Keywords:** Stereotactic biopsy, Diagnostic yield, Brain tumor, Sodium fluorescein, 5-aminolevulinic acid, Glioma

## Abstract

**Background:**

In patients affected by central nervous system tumors not amenable to surgical resection, stereotactic needle biopsy today represents the only available diagnostic approach. Achieving a high diagnostic yield is essential for timely therapeutic decision-making. This study investigates the impact of intraoperative fluorophores (sodium fluorescein and 5-aminolevulinic acid) use on the diagnostic accuracy of stereotactic brain biopsies.

**Methods:**

We retrospectively analyzed 516 stereotactic brain biopsies performed between 2015 and 2024. Data collected included fluorophore use, technique employed, histopathological and molecular results, and procedural outcomes. Diagnostic yield was defined as the proportion of conclusive diagnoses, in accordance with updated WHO classification criteria, while an extended definition, referred to as diagnostic success, included incomplete diagnoses; conversely, diagnostic failure was defined as inconclusive diagnosis. Statistical analyses included Chi-square testing and logistic regression.

**Results:**

Fluorophores were used in 314 procedures (60.9%), while 202 (39.1%) were done without (control group). The diagnostic yield was 76.1% with fluorophores versus 68.8% without. When including incomplete diagnoses (diagnostic success) the accuracy rose up to 93.3% in the fluorophore group versus 78.2% in the control group. Fluorophore use was associated with a 69.3% relative reduction in diagnostic failure (*p* < .001). Logistic regression confirmed that fluorophores significantly increased the odds of diagnostic success (OR = 3.89, *p* < .001), without significant histology-based selection bias.

**Conclusion:**

Intraoperative fluorophores application significantly enhances diagnostic yield in stereotactic brain biopsies, independently of tumor histology. These results support a wider implementation of fluorescence-guided biopsy techniques to improve diagnostic accuracy in neuro-oncology.

**Supplementary Information:**

The online version contains supplementary material available at 10.1007/s10072-026-09032-1.

## Introduction

In patients with central nervous system (CNS) tumors who are not eligible for surgical resection due to lesion eloquence, multifocality, age, comorbidities, or poor functional status, stereotactic biopsy (SB) often represents the only viable diagnostic strategy [1–6]. Despite its minimally invasive nature, the procedure is not free from risk and remains clinically useful only if it yields a conclusive diagnosis [7–11].

Over the last decade, advances in imaging, neuronavigation, and fluorescence-guided techniques allowed to increase the diagnostic yield (DY) of SB [12–17]. Achieving a reliable histomolecular diagnosis is now more important than ever, given that the latest World Health Organization (WHO) CNS tumor classification emphasize molecular profiling as an essential component of treatment planning [18–20]. However, a relevant percentage of biopsies still result in uncertain or non-diagnostic outcomes, which may delay therapy and adversely impact prognosis [21–23].

Among the strategies developed to improve needle biopsy accuracy, the intraoperative use of fluorophores such as sodium fluorescein (SF) and 5-aminolevulenic acid (5-ALA) has gained interest. These agents, initially introduced to assist tumor resection, are increasingly applied in SB to confirm the effective lesional target due to dye enhancement [24–35]. Yet, despite their promising rationale, current evidence on their real-world effectiveness remains limited, fragmented, and often exhibit considerable methodological heterogeneity.

The present study aims at addressing this gap by retrospectively analyzing a large cohort of patients who underwent stereotactic needle brain biopsy at a high-volume tertiary center. The primary aim is to quantify the impact of intraoperative fluorophore use on diagnostic success, and to explore whether specific tumor subtypes influence this association. Ultimately, the goal is to determine whether fluorescence-guided biopsy should be systematically adopted in neuro-oncological protocols, particularly for patients deemed inoperable, thus needing rapid adjuvant therapies.

## Materials and methods

### Study design

This is a retrospective comparative study designed to assess the diagnostic value of intraoperative fluorophore use during stereotactic brain biopsies. Data were collected from clinical records and structured into a dedicated database. Biopsies were performed using either a frame-based or frameless technique, based on surgeon preference and lesion location; intraoperative dye was selected upon histological suspicion and surgeon preference as well. Fluorophore administration and intraoperative visualization followed standard institutional protocols.

### Study population

**Fig. 1:**
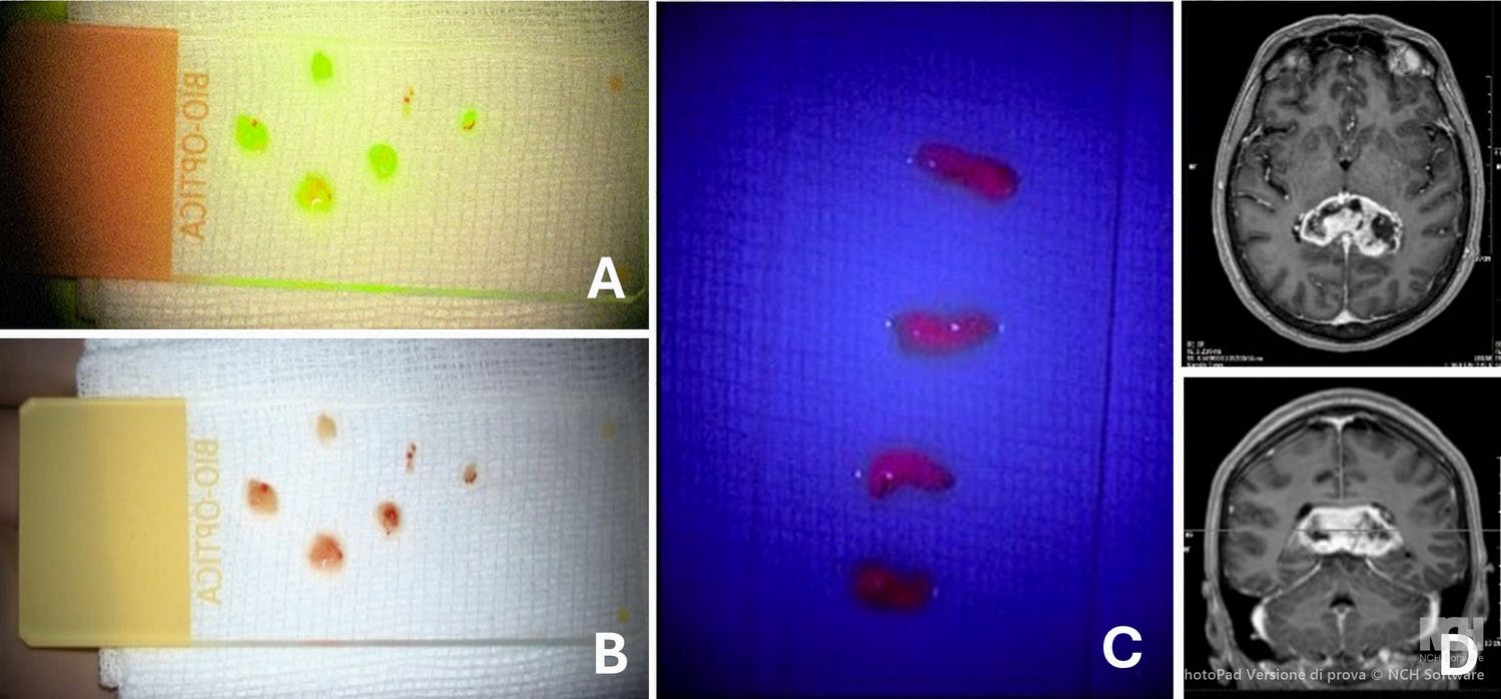
Fluorescein (5 mg/kg e.v.)-guided stereotactic biopsy of a high-grade glioma. Fluorescent under YE560 filter (**A**) and white-light (**B**) appearance of biopsy specimens. Biopsy specimens obtained under 5-ALA (20 mg/Kg orally)-guided fluorescence (**C**). Axial and coronal T1-weigthed with e.v. contrast administration Magnetic Resonance (MR) images of a patients with bilateral splenium of the corpus callosum glioblastoma who underwent stereotactic biopsy (**D**)

Patients who underwent stereotactic brain biopsy at the (Fondazione IRCCS Istituto Neurologico Carlo Besta, Milano, Italy) between January 2015 and December 2024 were included. Following an institutional multidisciplinary team (MDT) board discussion (Tumor Board), biopsies were offered to patients who were deemed not eligible for resection due to high surgical or patient-related risks, lesions located in eloquent brain areas and/or deep location, or multifocality (Fig. 1).

Inclusion criteria were the following: patients of any age or gender with radiological suspicion of CNS tumor requiring histopathological diagnosis via SB, following MDT tumor board discussion. Exclusion criteria were: inability to provide informed consent; previous history of anaphylaxis or allergy to SF or 5-ALA; Recent myocadiac infarction or stroke (< 90 days); Severe renal or hepatic failure; Pregnancy or lactation.

### Objective of the study and statistical analysis

The primary objective of the study was the calculation of the DY (i.e. conclusive diagnoses), the diagnostic success (DS) (any diagnosis, although incomplete, see below) or failure (i.e. inconclusive diagnosis).

Secondary objectives included the assessment of histology-based risk of failure and the impact of fluorophore use on biopsy performance (DY, DS or failure).

More in depth, DY was defined as the proportion of fully conclusive diagnoses with complete histological and/or molecular profiles. DS included both complete and uncertain diagnoses with/without WHO grade or without molecular profile but allowing for therapeutic management. Conversely, failures were all inconclusive diagnosis that precluded any therapy indication.

Subgroup analysis by tumor type was conducted. The impact of fluorophore use was quantified via absolute/relative differences in success rates. Chi-square tests assessed the associations. Binary and multinomial logistic regressions evaluated the predictors of success. Results were expressed as Odds Ratio (Ors) with 95% confidence interval. A *p* < 0.05 was considered significant. Analysis was conducted using IBM SPSS Statistics v29.

### Surgical procedure and fluorophore use

Briefly, biopsies were performed under general anesthesia (GA) with either frame-based or frameless guidance (Navigus™, iSYS1®, or AutoGuide™). Fluorophores were administered as follows:

- Sodium fluorescein 20% (*Fluoresceina Sodica 20%, Monico SPA, Venezia/Mestre Italy*) (5 mg/kg intravenously at GA induction or upon entrance in the operating room in case of awake procedures), according to literature indications by Acerbi et al. [31], following Determination 905/2015, Official Gazette No. 168, dated July 22, 2015. The specimens were then visualized using the YELLOW560 filter of the ZEISS PENTERO 900 or Kinevo microscopes (Karl ZEISS, Oberkochen, Germany).
- 5-ALA (20 mg/kg orally, 3–4 h before surgery) according to literature indications by Stummer et al. [36]. The specimens were then visualized using the BLUE400 filter of the ZEISS PENTERO 900 or Kinevo microscopes.

The biopsy target was selected with neuronavigation (Stealth Station S7 and S8, Medtronic, Minneapolis, USA). After dural opening, a side-cutting needle was advanced to the target. Samples (minimum of six) were collected until deemed sufficient by the surgeon.

Tissue samples were evaluated intraoperatively under the specific filter to assess for fluorescence uptake (Fig. 1). Its presence or absence was documented in the surgical report.

Samples were classified following WHO CNS criteria: 2007 (for samples before 2017), 2016 (2017–2021), and 2021 (2022 onward) [37–40].

Standard postoperative clinical and imaging follow-up was performed within 24 h from surgery. This included neurological examination, contrast-enhanced MRI or CT, and laboratory tests to assess complications or adverse events related to fluorophore administration.

## Results

### Patient demographics

Out of 524 cases, 516 tumor-related biopsies in 500 patients were included, as 12 patients underwent two biopsies and 2 patients underwent three. There were 176 females and 340 males. The mean age of all patients was 56 years with a range of 4–86 years (Std. deviation ± 15.708).

The lesions were superficial/lobar in 50.9% of cases and deep-seated in 49.1% of cases (Table 1).

**Table 1:** Location of 516 biopsies included in the study

Location	Frequency (%)	Absolute number
Superficial	50.9	263
Frontal	25.8	133
Parietal	10	52
Temporal	9.7	50
Cerebellar	3.3	17
Occipital	2.1	11
Deep	**49.1**	**253**
Thalamus	20	103
Corpus callosum	9.7	50
Peritrigonal	4.8	25
Basal Ganglia	3.9	20
Insular	3.3	17
Midbrain	1.9	10
Intraventricular	1.7	9
Pons	1.2	6
Medulla Oblongata	0.8	4
Hypothalamus	0.6	3
Capsular	0.4	2
Cingulate cortex	0.2	1
Lamina terminalis	0.2	1
Pineal gland	0.2	1
Sellar	0.2	1
Total	**100**	**516**

Among the 14 patients who required a repeated biopsy, 8 had initially undergone a procedure without fluorophore assistance.

In summary, the study included 366 gliomas, glioneuronal tumors, and neuronal tumors, 5 brain metastases, 67 cerebral lymphomas, 10 other tumors and conditions in the differential diagnosis, and 68 cases labeled as failed. A detailed report of all diagnoses is provided in Table 1 of the supplementary materials.

The use of dye during the procedures has evolved over time. 5-ALA has been used in 245 cases since its introduction in 2016. Fluorescein has been used since 2017, in a total of 69 cases. In the early years, the decision to use a dye varied from case to case. Since 2021, however, nearly all procedures have been performed with fluorophore assistance (Table 2).

**Table 2:** Distribution of dyes used in brain biopsies

Dye	Frequency (*n*)	Percent (%)
No dye	202	39.1
5-ALA	245	47.5
Fluorescein	69	13.4
*Total*	***516***	***100.0***

### Diagnostic outcome

When comparing the DY between procedures performed with and without the use of a fluorophore, a higher success rate was observed in the fluorophore group. Specifically, among the 314 cases in which a fluorophore was used, the DY (successful diagnoses) was 76.1%. In contrast, the DY without fluorophore use, based on 202 cases, was 68.8%. The proportion of failed diagnoses was markedly reduced in the fluorophore group (6.7%) compared to the non-fluorophore group (21.8%), while incomplete diagnoses accounted for 17.2% and 9.4%, respectively (Table 3).

**Table 3:** Crosstabulation between diagnosis and dye use

Outcome	With fluorophore (*n* = 314)	%	Without fluorophore (*n* = 202)	%	Total (*n* = 516)
*Successful diagnosis*	239	76.1%	139	68.8%	378
*Incomplete diagnosis*	54	17.2%	19	9.4%	73
*Failed*	21	6.7%	44	21.8%	65
*Total*	314	100%	202	100%	516

When DY was redefined to include both successful and incomplete diagnoses (DS) the overall yield raises to 73.3% (378/516 procedures): specifically, in the fluorophore group, 239 cases were classified as successful and 54 as incomplete, resulting in a combined DS rate of 93.3% (293 out of 314). In contrast, the non-fluorophore group had 139 successful and 19 incomplete diagnoses, corresponding to a total DS rate of 78.2% (158 out of 202). This expanded definition highlights an even more pronounced benefit associated with fluorophore use, not only in terms of reducing outright diagnostic failure but also in supporting partial diagnostic outcomes when full success is not achieved.

When comparing the two groups, fluorophore-assisted biopsies showed a 10.6% relative increase in DY (complete diagnoses only: 76.1% vs 68.8%), and an even greater 19.3% relative improvement in overall DS when including incomplete diagnoses (complete + incomplete diagnoses: 93.3% vs 78.2%). Additionally, the use of fluorophores resulted in a 69.3% relative reduction in diagnostic failures (6.7% vs 21.8%). These findings suggest that the use of a fluorophore may contribute to an improved DY and an even greater DS, while also reducing the failure rate.

A chi-square test of independence revealed a statistically significant association between the use of intraoperative dyes and the binary outcome diagnosis (χ^2^(successful) = 25.437, *p* < 0.001), suggesting that the distribution of outcomes varied according to dye use (Table 2, supplementary materials).

Specifically, patients who received a fluorophore were more likely to belong to the diagnosis/ successful group compared to those who did not receive any dye. To further explore this association, a binary logistic regression was conducted. The results confirmed that fluorophore use was a significant predictor of the outcome, with an odds ratio of 3.89 (OR = 3.885, 95% CI: 2.231–6.766, *p* < 0.001), indicating that patients who received a fluorophore had nearly four times greater odds of being in the diagnosis = successful category compared to those without dye (Table 3, supplementary materials).

Multinomial logistic regression analysis demonstrated that the use of fluorophore dye was significantly associated with an increased likelihood of achieving both complete and incomplete diagnoses, compared to diagnostic failure. Specifically, patients who received a fluorophore were 3.60 times more likely to obtain a complete diagnosis (OR = 3.603, 95% CI: 2.057–6.309, *p* < 0.001), and 5.96 times more likely to achieve an incomplete diagnosis (OR = 5.955, 95% CI: 2.849–12.447, *p* < 0.001), relative to those who underwent procedures without dye (Table 4, supplementary materials).

To assess the association between tumor histology and the use of intraoperative fluorophores we used a multinomial logistic regression. The analysis revealed that biopsies resulting in diagnostic failure were significantly more likely to have been performed without the use of a fluorophore (OR = 3.592, 95% CI: 1.754–7.357, *p* < 0.001). This suggests that fluorophore omission is strongly associated with diagnostic failure (Table 5, supplementary materials).

Therefore, fluorophore use appears homogeneous across tumor types, except in cases where the procedure results in diagnostic failure.

Finally, a subgroup analysis of the fluorophore group was performed. This showed that 5-ALA was mostly used in MRI enhancing lesions, while SF was used in a wider range of lesions, including those that did not enhance contrast on preoperative MRI. As a consequence, in the SF group we diagnosed more low grade gliomas (13.3% versus 3.3%), while the 5-ALA group resulted in a higher percentage of glioblastomas (42% versus 30.4%). Both 5-ALA and SF-aided biopsies resulted in a high grade glioma (around 35% each) diagnosis, thus likely including homogeneous and non-homogeneous contrast enhancing lesions (Table 4).

**Table 4:** Subgroup analysis of the *fluorophore group*

Histology	5-ALA	Sodium fluorescein
Glioblastoma	102 (42%)	21 (30.4%)
High grade glioma*	86 (35.5%)	24 (34.9%)
Low grade glioma	8 (3.3%)	9 (13%)
Lymphoma	30 (12.7%)	9 (13%)
Metastasis	4 (0.2%)	0
Failed	15 (6.3%)	6 (8.7%)
*total*	***245 (100%)***	***69 (100%)***

^*^ Including both complete and incomplete diagnosis (see text)

## Discussion

This study was developed to assess the fluorophore value during SB. While monocentric studies offer valuable preliminary insights, their small sample sizes often lack the statistical robustness and clinical generalizability required. Conversely, systematic reviews, despite analyzing larger populations by pooling multicenter data, are limited by substantial heterogeneity stemming from differences in patient selection, fluorophore type, imaging methods, and study designs, which compromises the interpretability of their conclusions [41, 42].

Despite the promising results seen so far, the clinical relevance of fluorophore use remains uncertain due to the significant heterogeneity across the available studies, particularly regarding methodologies, patient populations, and the correlation between fluorescence and histopathological findings. As a result, several authors have advocated for future prospective, randomized trials with standardized protocols, in order to ensure greater comparability and to validate the true utility of fluorophores in the setting of stereotactic needle brain biopsies [43].

In this study, by analyzing a larger and more representative patient population than most previously published monocentric studies, and by maintaining strict methodological consistency across all cases, the aim was to overcome both the limitations of sample size and the heterogeneity that have historically complicated the interpretation of results in this field.

The study included a cohort of 516 biopsies performed on 500 patients: 202 procedures were performed without the use of a fluorophore, while 314 were performed with fluorophore assistance, ensuring a consistent and comparable sample size for both groups.

Focusing on the biopsy outcome, the diagnostic yield was calculated according to the definition proposed by Khatab et al.: “the likelihood that a test or procedure will provide the information needed to establish a diagnosis which is certain and precise according to the WHO CNS tumor classification system” [21]. This differs from the more commonly used definition of DY as “the likelihood that a test or procedure will provide the information needed to establish a diagnosis” [44]

The Khatab definition was selected because, in the modern management of CNS tumors, molecular diagnosis has become crucial, providing essential information that directly influences prognosis, therapeutic decisions, and access to clinical trials. Despite technological advances allowing even small stereotactic biopsies to yield sufficient material for molecular profiling, diagnostic reliability remains a major concern.

In our cohort, DY was calculated separately for the no-fluorophore and fluorophore-assisted groups, with a DY of 68.8% in the first group and 76.1% in the second, corresponding to a 10.6% relative increase in DY.

In this study, “diagnostic yield” refers exclusively to complete diagnoses, while “diagnostic success” includes both complete and incomplete diagnoses. This terminological distinction is maintained to ensure clarity when interpreting internal results and comparing them with previous studies.

Furthermore, the rate of diagnostic failures dropped from 21.8% to 6.7%, corresponding to a 69.3% relative reduction.

It is important to highlight that the apparent increase in incomplete diagnoses with fluorophore use (17.2% vs 9.4%) does not represent a detrimental effect of fluorophores. Instead, this difference is attributable to the introduction of the "incomplete diagnosis" category following the 2021 WHO CNS classification update, during a period when fluorophore use had already been routinely implemented at our institution. The lower rate of incomplete diagnoses in the no-fluorophore group is mainly because most of these cases were diagnosed before the new classification system was adopted.

The "incomplete diagnosis" group includes cases where a histological categorization was achieved but molecular markers were missing.

One way to bypass this issue would be to apply the classical DY definition, considering only two diagnostic outcomes: successful or failed. Using this method, the overall diagnostic success would increase to 93.3% in the fluorophore group compared to 78.2% in the non-fluorophore group, resulting in a 19.3% relative improvement.

Nevertheless, a true assessment of the impact of fluorophores on incomplete diagnoses would require a prospective randomized study. However, given the significant reduction in diagnostic failures and the evident clinical benefit associated with fluorophore use, such a study would likely not be ethically acceptable.

To further investigate the relationship between fluorophores use and biopsy outcomes, we did not limit our analysis to a simple description of the results. Instead, we applied more complex statistical methods to assess the true correlation between these two variables. Statistical analysis revealed a strong association between the use of intraoperative fluorophores and DS. Specifically, patients who received a fluorophore were significantly more likely to achieve a successful diagnosis compared to those who did not. This finding was initially confirmed by a chi-square test (χ^2^ = 25.437, *p* < 0.001) and further supported by binary logistic regression analysis, which showed that the use of a fluorophore nearly quadrupled the odds of obtaining a successful diagnosis (OR = 3.89, 95% CI: 2.231–6.766, *p* < 0.001).

Concerning the type of fluorophore, the attending surgeon was free to choose the one deemed more appropriate for each case. Broadly speaking, our center attitude is the following: when the target lesion enhances contrast on preoperative MRI both fluorophores can be used; conversely, if the target lesion does not enhance or it enhances partially/not homogenously we suggest using SF. Consequently, 5-ALA was used more often when the preoperative suspicion was a high grade glioma/glioblastoma, while SF was used in a wider range of lesions, including those that did not enhance contrast on preoperative MR. This was confirmed by the subgroup analysis of the fluorophore group (Table 4).

Another important finding to discuss is the use of fluorophores across different CNS tumor types. While most studies focus on the application of dyes in high-grade tumors, this study aimed to assess whether any differences in fluorophore performance could be observed across a broader range of tumor types. Tumors were categorized into four main groups: glioblastomas, diffuse large B-cell lymphomas, astrocytomas, and a heterogeneous group comprising all other CNS tumor findings. A multinomial logistic regression was then performed: the analysis showed that the diagnostic performance of fluorophores appeared consistent across the different tumor categories, suggesting that fluorescence provided equally useful intraoperative information regardless of tumor histology. However, biopsies resulting in diagnostic failure were significantly more likely to have been performed without fluorophore assistance (OR = 3.592, 95% CI: 1.754–7.357, *p* < 0.001), indicating that the absence of fluorescence signal is strongly associated with an increased risk of diagnostic failure.

Taken together, these findings reinforce the role of intraoperative fluorophores in enhancing the DY of stereotactic brain biopsies across a wide spectrum of CNS lesions. These results are consistent with the existing knowledge in the field of “open” resective neurosurgery, where the use of intraoperative fluorophores has already been shown to improve the identification of pathological tissue and to enhance surgical outcomes.

Overall, the presented data suggest that the use of fluorophores can significantly improve diagnostic reliability and may contribute to optimizing patient management in neuro-oncology.

Despite the significantly increased DY associated with the use of intraoperative fluorophores, our results also highlight the persistence of a non-negligible percentage of inconclusive biopsies. This residual diagnostic uncertainty, especially when molecular analyses fail or tissue is insufficient, urges the exploration of additional intraoperative strategies aimed at improving sampling precision.

## Limitations

Despite its promising results, this study has some limitations. First, both fluorescein and 5-ALA were used upon surgeon’s preference, introducing variability in fluorescence interpretation and possible biases. Second, the 10-year study period spanned three editions of the WHO CNS classification, affecting diagnostic categorization, particularly with the recent concept of “incomplete diagnosis.” Lastly, its retrospective, single-center design may limit generalizability and carries a potential risk of selection bias.

## Conclusion

Stereotactic needle brain biopsies are a cornerstone in the diagnosis of CNS tumors and represent the best available basis for neuro-oncological management in patients who are not eligible for surgical gross total resection.

In this context, achieving a high diagnostic yield is crucial for optimizing patient care and treatment planning.

This study demonstrates that the use of intraoperative fluorophores significantly increases the DY and reduces the rate of diagnostic failures in a large, methodologically consistent patient cohort.

These findings are consistent with the established benefits of fluorophores observed in “open” resective neurosurgery and support their broader adoption also in the setting of SB.

Although further research in more homogeneous cohorts is desirable to consolidate these results, they provide strong support for the clinical value of fluorescence-guided stereotactic biopsy.

## Supplementary Information

Below is the link to the electronic supplementary material.

Supplementary file1 (DOCX 26 KB)

## Data Availability

The data presented in this study are available upon request to the corresponding author.
