# Supplementary material for: Fluorescence-aided stereotactic needle biopsies for brain tumors: experience on 516 cases
Source: Neurol Sci. 2026 Apr 15;47(5):419. doi: 10.1007/s10072-026-09032-1 (PMC13079475; doi:10.1007/s10072-026-09032-1)
Supplement: Supplementary file 1 — Supplementary file1 (DOCX 26 KB) [file 10072_2026_9032_MOESM1_ESM.docx]

**Table 1 supplementary materials:** Distribution of diagnoses in the study cohort.

| ***Diagnosis*** | ***Frequency*** | ***Percent*** |
| --- | --- | --- |
| Anaplastic astrocytoma (WHO 3) | 10 | 1.9 |
| Anaplastic astrocytoma (WHO 3) | 29 | 5.6 |
| Anaplastic astrocytoma (WHO 4) | 2 | 0.4 |
| Anaplastic astrocytoma, ill-mutated (WHO 3) | 3 | 0.6 |
| Anaplastic astrocytoma (WHO 3) | 2 | 0.4 |
| Anaplastic ependymoma (WHO 3) | 2 | 0.4 |
| Anaplastic ganglioglioma (WHO 3) | 1 | 0.2 |
| Anaplastic oligodendroglioma | 1 | 0.2 |
| Anaplastic oligodendroglioma | 1 | 0.2 |
| Anaplastic oligoastrocytoma | 1 | 0.2 |
| Astrocytoma, IDH-mutated pleomorphic (WHO 3) | 1 | 0.2 |
| Astrocytoma IDH-mutated (WHO 2) | 1 | 0.2 |
| Astrocytoma IDH-mutated (WHO 3) | 1 | 0.2 |
| Astrocytoma IDH-mutated (WHO 4) | 1 | 0.2 |
| Astrocytoma IDH-mutated | 1 | 0.2 |
| Astrocytoma with initial anaplasia | 3 | 0.6 |
| Astrocytoma, IDH-mutated (WHO 2) | 2 | 0.4 |
| Astrocytoma, IDH-mutated (WHO 3) | 1 | 0.2 |
| Astrocytoma, IDH-mutated NOS | 1 | 0.2 |
| CNS embryonal tumor, adamantomatous (WHO 1) | 56 | 10.9 |
| Craniopharyngioma, adamantinomatous (WHO 4) | 11 | 2.1 |
| Desmoplastic nodular medulloblastoma (WHO 4) | 1 | 0.2 |
| Diffuse large B cell lymphoma (CD20+) | 68 | 13.2 |
| Diffuse large B cell lymphoma, H3-K27 altered (WHO 4) | 1 | 0.2 |
| Epitheloid Glioblastoma (WHO 4) | 2 | 0.4 |
| Failed | 5 | 1.0 |
| Fibrillary astrocytoma | 1 | 0.2 |
| Ganglioglioma | 1 | 0.2 |
| Gemistocytic astrocytoma (WHO 2) | 30 | 5.8 |
| Germinoma | 1 | 0.2 |
| Giant cell Glioblastoma (WHO4) | 143 | 27.7 |
| Glioblastoma (WHO4) | 5 | 1.0 |
| Glioma | 1 | 0.2 |
| Glioneuronal tumor | 1 | 0.2 |
| Gliosis | 1 | 0.2 |
| H3-K27 Altered Diffuse Midline gliomas | 7 | 1.4 |
| H3-K27 Altered Diffuse Midline gliomas (WHO 4) | 8 | 1.6 |
| High grade glioma | 54 | 10.5 |
| High grade glioma, IDH-mutated | 1 | 0.2 |
| High grade glioma, H3-wildtype, BRAF (V600E) mutated | 1 | 0.2 |
| Hodgkin lymphoma | 1 | 0.2 |
| Low grade glioma, high grade glioma, IDH-wildtype | 4 | 0.8 |
| Lymphoplasmacytic lymphoma | 1 | 0.2 |
| Marginal infiltration of glial neoplasm | 3 | 0.6 |
| MALT lymphoma | 1 | 0.2 |
| Metastasis from epithelial neoplasm | 1 | 0.2 |
| Mix carcinoma | 1 | 0.2 |
| Mtx therapy | 1 | 0.2 |
| NEC (not elsewhere classified) | 16 | 3.1 |
| Neoplasm | 1 | 0.2 |
| Oligoastrocytoma | 1 | 0.2 |
| Oligodendroglioma | 3 | 0.6 |
| Oligodendroglioma IDH-mutated | 1 | 0.2 |
| Oligodendroglioma IDH-mutated (WHO 2) | 1 | 0.2 |
| Oligodendroglioma IDH-mutated (WHO 2/3) | 1 | 0.2 |
| Oligodendroglioma IDH-mutated, and 1p/19q-codeleted | 3 | 0.6 |
| Pilocytic astrocytoma | 5 | 1.0 |
| Pineoblastoma (WHO4) (Ki67 50%) | 2 | 0.4 |
| Xanthoastrocytoma pleomorphic | 1 | 0.2 |
| Xanthoastrocytoma pleomorphic (WHO2) | 1 | 0.2 |
| **Total** | **516** | **100.0** |

**Table 2 supplementary materials:** Chi-Square Tests for association between dye usage and diagnostic yield.

| ***Test*** | ***Value*** | ***df*** | ***Asymptotic Significance (2-sided)*** | ***Exact Sig. (2-sided)*** | ***Exact Sig. (1-sided)*** |
| --- | --- | --- | --- | --- | --- |
| **Pearson Chi-Square** | 25.437^a^ | 1 | <0.001 | - | - |
| **Continuity Correction^b^** | 24.085 | 1 | <0.001 | - | - |
| **Likelihood Ratio** | 24.849 | 1 | <0.001 | - | - |
| **Fisher's Exact Test** | - | - | - | <0.001 | <0.001 |
| **N of Valid Cases** | 516 | - | - | - | - |

a. 0 cells (0.0%) have expected count less than 5. The minimum expected count is 25.45.

b. Computed only for a 2x2 table.

**Table 3 supplementary materials:** Binary logistic regression on the effect of fluorophore use on diagnosis.

| ***Step^a^*** | ***B*** | ***S.E.*** | ***Wald*** | ***df*** | ***Sig.*** | ***Exp(B)*** | ***95% CI for Exp(B) Lower*** | ***95% CI for Exp(B) Upper*** |
| --- | --- | --- | --- | --- | --- | --- | --- | --- |
| **Diagnosis** | 1.357 | 0.283 | 23.001 | 1 | <0.001 | 3.885 | 2.231 | 6.766 |
| **Constant** | 1.278 | 0.170 | 56.247 | 1 | <0.001 | 3.591 | - | - |

a. Variable(s) entered on step: diagnosis

Binary logistic regression results showed that the use of fluorophore dye was a significant predictor of diagnostic success. Patients who received a fluorophore were 3.89 times more likely to achieve a diagnostic success compared to those who received a fluorophore were 3.89 times more likely to achieve a diagnostic success compared to those who received no dye (OR = 3.885, 95% CI: 2.231–6.766, p < .001).

**Table 4 supplementary materials:** Multinomial logistic regression on the effect of fluorophore use on diagnosis.

| ***Categoryᵃ*** | ***Predictor*** | ***B*** | ***Std. Error*** | ***Wald*** | ***df*** | ***Sig.*** | ***Exp(B)*** | ***95% CI for Exp(B)***  ***Lower-Upper*** |
| --- | --- | --- | --- | --- | --- | --- | --- | --- |
| **Diagnosis** | *Intercept* | 1.150 | 0.173 | 44.221 | 1 | <0.001 | – | – |
|  | *[Dye = fluorophore]* | 1.282 | 0.286 | 20.100 | 1 | <0.001 | 3.603 | 2.057 – 6.309 |
|  | *[Dye = no dye]* | 0ᵇ | – | – | – | – | – | – |
| **Incomplete diagnosis** | *Intercept* | -0.840 | 0.275 | 9.358 | 1 | 0.002 | – | – |
|  | *[Dye = fluorophore]* | 1.784 | 0.376 | 22.498 | 1 | <0.001 | 5.955 | 2.849 – 12.447 |
|  | *[Dye = no dye]* | 0ᵇ | – | – | – | – | – | – |

a The reference category is: failed.

b This parameter is set to zero because it is redundant.

Multinomial logistic regression showing that fluorophore use significantly increased the odds of both complete (OR = 3.603) and incomplete (OR = 5.955) diagnosis versus diagnostic failure (p < .001).

**Table 5 supplementary materials:** Multinomial logistic regression analysis of the association between tumor type and fluorophore use**.**

| ***Dyeᵃ*** | ***B*** | ***Std.Error*** | ***Wald*** | ***df*** | ***Sig.*** | ***Exp(B)*** | ***95% CI for Exp(B) [Upper-Lower]*** |
| --- | --- | --- | --- | --- | --- | --- | --- |
| *No-dye group* |  |  |  |  |  |  |  |
| **Intercept** | -0.495 | 0.254 | 3.801 | 1 | .051 | – | – |
| **DLBCL** | 0.258 | 0.352 | 0.538 | 1 | .463 | 1.295 | 0.649 – 2.582 |
| **Other tumors of CNS** | -0.143 | 0.345 | 0.173 | 1 | .678 | 0.866 | 0.441 – 1.703 |
| **Glioblastoma** | -0.181 | 0.300 | 0.366 | 1 | .545 | 0.834 | 0.463 – 1.502 |
| **Failed** | 1.279 | 0.366 | 12.227 | 1 | <.001 | 3.592 | 1.754 – 7.357 |
| **High-grade glioma** | -0.673 | 0.398 | 2.865 | 1 | .091 | 0.510 | 0.234 – 1.112 |
| **Astrocytoma** | 0ᵇ | – | – | 0 | – | – | – |

a The reference category for the dye variable is: DLBCL.

b This parameter is set to zero because it is redundant.
